# Visceral Neurosis (Neuralgia)

**Published:** 1897-04

**Authors:** Byron Robinson

**Affiliations:** Chicago


### TEXAS MEDICAL JOURNAL.

ESTABLISHED JULY, 1885.

PUBLISHED MONTHLY.—SUBSCRIPTION $2.00 A YEAR.

VOL. XII. AUSTIN, APRIL, 1897. NO. 10.

### Original Contributions.

For the Texas Medical Journal.

### VISCERAL NEUROSIS.

(Neuralgia.)

BYRON ROBINSON, B. S., M. D., CHICAGO.

IN VISCERAL NEUROSIS or neuralgia we are doubtless dealing with a peculiar form of malnutrition of the nerves of sensation. Hence in these days of scalpel or no scalpel, of sweeping removal or surgical repair, it behooves us to diagnose with cauthe symptoms of disease. In disease we are seldom dealing not only with signs which are distinct clues to disease, but chiefly with symptoms which are only indications of pathology. In visceral (abdominal) neurosis, we are dealing with organs which possess (a) motion, (b) sensation and (c) secretion, i. e., such organs have muscles which are set in motion by motor nerves, sensation made manifest by some irritation on the sensory nerve ends and secretion which proceeds normally in certain quantities, but in disturbed conditions (a) excessive, (b) deficient or (c) disproportionate.

In visceral (abdominal) neurosis we are chiefly dealing with the sympathetic—a nerve of rhythmical motion and dull sensation. The term visceral (abdominal) neurosis is a mere name of a symptom in the mind of many physicians, as we say the kettle boils when we really mean that the water boils, or is raised to such a degree of temperature that the ebulition occurs in the water.

Visceral neurosis indicates that some deep condition, assimilation or vicious process is proceeding somewhere. The observing physician of experience commonly associates in his mind visceral neurosis with some debilitating process in age or sex. We cast about for predisposing causes, and examine them as a neurotic temperament, hereditary or acquired. One can acquire a neurotic disposition by dissipation, sexually, with narcotics, by excessive and prolonged labor, the absorption of poisonous substances as lead, arsenic or phosphorus. Rapid changes of temperature brings on visceral neuralgia. (b) We also take into account sex. It is difficult to say which sex suffers the most from visceral (abdominal) neurosis. I should judge women do. But different varieties of visceral neurosis prevail in each sex, and at different periods of life.

(c.) The chief age of visceral neurosis is from 20 to 60. Few cases occur before 20 and rarely after 60. (d) The sexual life of woman is rich in visceral (abdominal) neurosis at different periods as 1, at puberty; 2, at the menopause; 3, at the mentrual period; 4, during pregnancy; 5, in the puerperium; 6, there are neuroses from excess or abstinence of venery. In the above six factors the circulation plays an important role. In short, the neuralgia is secondary to some other process. (e) Visceral (abdominal) neurosis or neuralgia is commonly associated with general malnutrition as in anæmia, cachexia from malignant disease, chlorosis, debility, mental or physical, from irritation, over strain. Diabetic, gouty and rheumatic persons suffer from visceral neuralgia. In the above factor irritation plays the chief role.

(f) In the ætiology of visceral neuralgia, we must include all kinds of trauma to nerves, contraction of cicatrical tissue, pressure of adjacent organs, tumors and tissue on nerves, adjacent inflammatory tissue. Dislocated organs dragging, as in visceral ptosis. In short trauma, pressure and dragging.

(g) Many visceral neuralgias rest on infection or intoxication, as malaria, typhoid fever, lead, copper, mercury and other agents.

(h) Catching cold (rapid changes of temperature) cold and wet weather play a role in the ætiology of visceral neuralgia.

(i) Visceral neurosis may depend on 1, a small abnormal brain; 2, deficient blood supply; 3, continued disease; 4, early senility; 5, temporary invagination of the bowels.

(3) A peculiar affection of the rectum of a neuralgic character sometimes arises. It occurs in robust as well as neurotic persons. The patient will go to bed well, and wake up at any hour of the night with a severe pain in the rectum about the large prostatic plexuses of man, and about the cervico-uterine ganglia of woman. I know one patient who has had such an affection for over ten years. The pain rises to a maximum and remains intense, grawing and grinding for from ten minntes to nearly an hour, when it will suddenly pass away. No cause can be assigned in this case, for the patient lives in apparently perfect health.

The symptoms, par excellence, of visceral neurosis is pain. The patients describe the pain in manifold ways, as boring, dragging, burning, stabbing, pressing, lancinating, grinding and tearing. Usually the pain is paroxysmal, ceasing in the intervals. The pain on lessening may be very irregular, slight or intense.

One point in neuralgia (visceral or otherwise) I doubt, and that is that the nerves have distinct, local points of tenderness. Dr. Vallerix announcement for example, the three tender points on the intercostal nerves. But by careful examination, and an opportunity to compress the nerves we would likely elicit pain in any or all points of a neuralgic nerve. The patient can scarcely give distinct localities of tenderness, for mechanical pressure elicits distinct pain. The irregularity of the various localities of pain in visceral neuralgia shows that it is not a mere local disorder, but some germinal malnutrition of the sensory apparatus. Visceral neuralgia not only occurs in the trunks but branches of nerves. As some patients will complain of pain in various regions of the hypogastric trunks, but irregular pain in the spermatic branches or in the testicle. During the attacks of visceral neuralgia, various accompanying secondary affections arise, as vasomotor disturbances, muscular disturbances. The vessels contract, lessening the amount of blood passing through them and muscular action brings on muscular contractions (colic) in local and remote regions of the abdomen, shifting, colicky, cramping pains characterize the visceral neuralgia. In one patient, on whom we operated the second time, complained of varying pains in the right side, we found the liver and stomach proiapsed considerably. Since the operations, she compains of irregular pains still in the right side where we made no interference. We did not operate for pain in the right side, but for other reasons, yet we noted much visceral ptosis of the stomach and liver in the region of these neuralgic pains. In many cases I have noted the evil effects of peritoneal adhesions previous and subsequent to abdominal section, and Dr. Lucy Waite and I have now operated on about a dozen patients a second time for the pain caused by peritoneal adhesions, fixing movable viscera and interfering with their function and peristalsis. Peritoneal adhesions produce as symptoms a kind of visceral neurosis or neuralgia; however, the pain of peritoneal adhesions is certainly more constant in the language of the patient, as dragging sensations, repeating itself on prolonged efforts. Another patient complained of a varying pain along the left ovarian plexus, and again for months in the region of the left kidney. Physically nothing could be discovered except that she was very anæmic. I am thoroughly convinced that considerable visceral pain arises from pressure of fæcal masses as they pass over the nerve plexuses, also that the hard irriating fæcal masses stir up local bowel contractions (colic) as they move toward the rectum. This accounts for the clinical fact that the visceral neuralgia pains fast disappear when cathartics are so used as to regulate a daily stool. In my practice of gynecology nothing has produced better results in constipation than the drinking of a full glass of water and with half a teaspoonful of epsom salts on retiring to bed, and going to stool promptly after breakfast every morning. The more I practice gynecology and abdomonal surgery, the more I become acquainted with visceral ptosis and its evil results, and the more I am convinced that visceral neuralgia has a physical basis whose pathology will become more manifest with study.

It is difficult to point out precisely symptoms of visceral neuralgia for the very simple fact that we do not yet know the definite functions of the visceral nerves. We must compare the visceral neuralgia with the better known neuralgia of the trigerminus. It has been stated that neuralgia is a prayer of the nerve for nourishment or of fresh blood. We often notice that a nerve subject to neuralgia is sensitive to pressure. So in our diagnosis we must follow the track of sensitive nerves in the abdomen. To do this we must know that there are great bundles or trunks of nerves called plexuses, which follow quite generally large blood vessels. Great ganglia exist in different localities of the abdomen which space forbids even naming. In short, we have to deal with the abdominal branch, the inferior mesenteric ganglion, the cervico-uterine ganglia and the lateral chain of ganglia and hosts of smaller ones—all connected by nerve cords. The sympathetic nervous system which supplies the abdominal viscera is partly independent from the remainder of the nervous system, and partly intimately connected with its ganglia by fibres from the brain and cord. The ganglia and fibers are the greater part motor and innervate the involuntary muscles of the viscera. We deal with the nervous system of the abdomen, as composed of the (a) lateral chain of ganglia; (b) the abdominal and pelvic splanchnics; (c) the rami communicantes; (d) the vagi nerves and (e) the abdominal branch with all the nerve ganglia. We have but space to mention the special forms of neuralgia, which have been attached to different abdominal organs under the general term of visceral neuralgia. Some of the following forms of visceral neuralgia have gained a place in medical literature.

1. Hepatic neuralgia or colica hepatica non-calculosa.
2. Neuralgia of the stomach or gastralgia.
3. Enteralgia (colica mucosa Nothnagel).
4. Ovarian neuralgia.
5. Neuralgia rectalis.
6. Neuralgia renalis.
7. Tubal colic.
8. Uterine neuralgia.

Hepatic neuralgia rests in the view that pain of a neuralgic character arises in the liver region when gall-stones do not appear in the stool, nor are found in the autopsy. Andral Budd, Freidrichs, Furbinger Durand, Fardel and Schuppel are names representing belief of hepatic neuralgia with no calculus as a cause. Gastralgia has been so long in medical literature that it need not be supported by any names. Enteralgia in its various indefinite forms is seen by gynecologic practitioners frequently.

Ovarian neuralgia is a disease glibly talked about, but very difficult to diagnose. I have listened perhaps hundreds of times to descriptions of patients suffering what some would designate ovarian neuralgia. Yet women do have irregular pain, slight and intense, in the ovaria. The ovary will be found sensitive and painful on pressure. It is the opinion of the writer that so-called ovarian neuralgia is a secondary process, and yet it doubtless exists as certain as neuralgia of the upper division of the trigeminus. Neuralgia of the rectum has a definite existence It comes and goes with great irregularity, arising chiefly a night and appears in persons of apparently robust heal th.

Neuralgia of the kidney rests on the fact that pain occurs in the region of the kidney, the kidney is sensitive to pressure, and no stone has been found in the kindey at the autopsy. The pain has been so severe that nephrectomy was performed, but the kidney contained no stone. Tubal and uterine colic, or so-called neuralgia rest on their peculiar structure. Their involuntary muscular walls being supplied by sympathetic nerves are liable to be set in motion by various forms of irritation, and hence from tonic and clonic spasms of their walls are liable to give rise to irregular flying pains or visceral neuralgia.

From a careful study of visceral neuralgia, it is evident that it is a secondary disease. It consists of a peculiar malnutrition of a sensitive nerve apparatus. The treatment of visceral neuralgia consits in improving nutrition, to relieve present distress by harmless means, and remove all depressing causes.

